# The effects of nearby trees on the positional accuracy of GNSS receivers in a forest environment

**DOI:** 10.1371/journal.pone.0283090

**Published:** 2023-03-15

**Authors:** Taeyoon Lee, Pete Bettinger, Krista Merry, Chris Cieszewski

**Affiliations:** Warnell School of Forestry and Natural Resources, University of Georgia, Athens, GA, United States of America; Taylor’s University - Lakeside Campus: Taylor’s University, MALAYSIA

## Abstract

Global Navigational Satellite System (GNSS) technologies are actively being developed to address the demand for enhanced positional accuracy. Smartphones are the most prevalent GNSS receiver today and have garnered attention thanks to improved positional accuracy and usability that can be accessed at an affordable price. In a forested environment, multipath error can deteriorate the positional accuracy, depending on the state of nearby vegetation. Therefore, this study was conducted to investigate the impacts of the size and location of vegetation on positional accuracy of GNSS receivers to determine whether the errors observed are systematic. Twenty-six control points within the Whitehall Forest GPS Test site in Athens, Georgia were used to evaluate positional accuracy of three different GNSS receivers (two traditional handheld GNSS receivers (including Garmin and Trimble receivers) and a smartphone). Thirty-five forest variables were developed from information around each control point to conduct a correlation analysis with observed horizontal position error in the positions determined by each device. In this study, we confirmed that the positional error of the smartphone was significantly lower than the Garmin receiver, and similar, but significantly different than the positional error observed by the Trimble receiver. It was confirmed that correlations between forest variables and horizontal position error regardless of the GNSS receiver employed were significant, yet trends were not consistent. The effect of the size of nearby trees on horizontal position error could not be generalized; however, the location of nearby trees on horizontal position error could.

## Introduction

Since the United States (NAVSTAR GPS) and Russian (GLONASS) global positioning systems began, Global Navigational Satellite Systems (GNSS) have developed relatively rapidly over the last 50 years. Today, GNSS constellations include NAVSTAR GPS, GLONASS, Galileo (European Union), BeiDou (China), NAVIC (India), and QZSS (Japan) and when combined, they currently have over 120 satellites in use for navigational purposes above Earth. These GNSS constellations utilize a variety of electromagnetic frequencies to transmit signals to GNSS receivers. These options facilitate many precise-positioning applications (such as navigation, timing, and remote sensing) through improved satellite visibility and ambiguity resolution of signals [1, 2]). Advancements in associated GNSS technologies (GNSS antenna chipsets and augmentation systems) have also acted to enhance the accuracy of horizontal positions determined by GNSS receivers.

GNSS receivers are used by people to navigate and map land and water features and were formally categorized as belonging to three grades (e.g., survey-, mapping-, and consumer-grade) depending on their built purposes (general ability to correctly determine the position and their general cost). However, this categorization of GNSS receivers into grades is becoming obsolete as GNSS technologies have advanced [3]. Indeed, new developments in GNSS technologies have made it possible to use lower cost, mass market GNSS receivers to obtain relatively accurate (2–10 m) horizontal position information [2]. In addition, the positional accuracy of lower grade receivers can be improved by using them in conjunction with other technologies such as RFID (radio frequency identification), UWB (ultra-wideband), and localization techniques such as INS (inertial navigation system) and RTK (real-time kinematic) [4–7]. Although augmentation through the aid of these advances in technology is not commonly employed in the forestry profession [6],. lower grade receivers are more generally used for navigation and mapping purposes in the southern United States [8].

There are a number of compact, low-cost, single or dual frequency GNSS receivers on the market today. Recently, smartphones have attracted the attention of researchers and forestry practitioners as an alternative GNSS receiver because they can address user desires for lower-cost, more portable, wearable devices [2, 7, 9, 10] that facilitate alternative uses (telecommunication, applications). About 331.2 million smartphones were shipped to people around the world during the third quarter of 2021, and the demand is expected to continue to be strong despite supply chain issues associated with the Covid-19 pandemic [7, 11, 12]. Some have therefore suggested that the smartphone should be considered as the most prevalent, general type of GNSS receiver in the market today [2, 13].

With the massive popularity of smartphones among society, the need to evaluate the feasibility of using them as GNSS receivers to collect positional information is important. Studies concerning smartphone positioning accuracy have significantly increased in the past few years with the development of mass-market GNSS chipsets and the accessibility of raw GNSS measurements [2, 10]. Low-cost, mass-market GNSS chipsets within smartphones as well as chipsets that can accommodate dual frequency multi-constellation signals, allow one to track modernized GNSS signals (i.e., L5/E5) [2]. These advances may facilitate enhanced positional accuracy by potentially mitigating ionospheric effects on signal propagation [7, 13]. With the advent of the Application Programming Interface 24 (API 24) in 2016, raw GNSS measurements including pseudoranges, carrier phases, Doppler GNSS measurement, navigation messages, and hardware clocks may be accessible to users and developers [2, 13]. This information would then allow one to investigate the potential augmentation improvements of various post-processing algorithms that have been designed for higher grade GNSS receivers [13]. However, these augmentations are generally beyond the reach of typical natural resource management professionals.

According to previous studies, the GNSS chipset typically equipped within smartphones has a few unique characteristics compared to the GNSS chipsets installed in other GNSS receivers. It seems that smartphones have been designed to be sensitive to weaker GNSS signals compared to other GNSS receivers, and smartphones may be able to utilize an Assisted GPS (A-GPS) technology, which uses the Internet or Wi-Fi signals to acquire satellite information, such as the almanac and satellite-specific ephemeris, from network providers [10, 13–16]. This additional information may be advantageous to the use of smartphones, by reducing the signal search space and the time required to determine a position, and this information can potentially increase positional accuracy even in indoor conditions [13]. However, smartphones may also have inherent weaknesses, as some employ duty cycling, which makes the satellite signal data collection process within smartphones operate in a discontinuous manner [13, 15]. This function was designed to prevent an adverse rate of battery drainage, but it causes discontinuities in phase observables, which can negatively affect the ability of a smartphone device to precisely determine a position [17]. Due to this and perhaps other hardware and software limitations, smartphones generally have a lower carrier-to-noise ratio (C/N_0_) when collecting positional information, compared to traditional GNSS receivers [2, 18, 19].

To overcome these limitations in obtaining high quality positional accuracy, many investigations into accuracy improvement have been conducted using smartphones. Yoon et al. [15] categorized these as: (a) hardware add-on or modification and (b) user-developed software efforts. For instance, an external geodetic grade antenna can be attached to a smartphone (as it can for other GNSS receivers) to acquire signals from satellites, rather than utilizing the embedded GNSS antenna within smartphones [2]. Adding an external geodetic grade GNSS antenna was considered a practical way to improve the positional accuracy determined by smartphones until the raw GNSS measurements were available [10, 15]. After users and developers could access the raw GNSS measurements, studies have mainly focused on developing algorithms or software to adjust positions arising from various error sources [2]. For instance, Yoon et al. [15] developed a DGNSS coordinate projection method which improved positional accuracy by 30–60% even without accessing raw GNSS measurements. In addition, various kinds of algorithms have been developed to help attain sub-meter or centimeter level positional accuracy [13]. Since the majority of studies have focused on attaining higher quality positional accuracy, they often use longer observation times (from an hour to over 40 hours) to determine a single position and they often evaluate the raw GNSS measurements. One study examined the positional accuracy of smartphones using shorter observation times (10 minutes), where sub-meter positional accuracy in open conditions were obtained with the aid of post processing [13]. However, spending 10 minutes to acquire sub-meter positional accuracy per location is not practical for mapping every tree (perhaps 100 or more) within a research study site or in the course of collecting an inventory of data that would support density-dependent growth and yield modeling efforts. Further, sub-meter positional accuracy was not achievable under a forest canopy cover condition with 10 minutes of observation time, even with the aid of post processing [13].

Multipath may be the largest source of error when any GNSS receiver is used within a forest [20]. Some studies have tried to reduce the impact of multipath error by using an anechoic chamber, a choke ring antenna, or a radio frequency shield box or plate [13, 17, 20–22]. However, it is almost impossible to mitigate the impact of multipath error when commonly navigating or working in a forest, as the arrangement of nearby obstructions (trees) and the arrangement of the satellite constellation are constantly changing as one moves around. Because of this, most studies (except [20]) have been conducted under open area or low multipath conditions to reduce the multipath error and attain the highest positional accuracy. There have been attempts to investigate the effects of nearby vegetation (forest age, composition, and juxtaposition of trees) on positional accuracy determined by GNSS receivers, yet only weak correlations with horizontal positional accuracy were observed [23–25]. Furthermore, none of these studies involved the use of smartphones. Tomaštík and Varga [13] assumed that the distance of a tree stems to the position being determined by a GNSS receiver would hinder GNSS signal reception as trees were closer to the receiver. Therefore, one can argue that the impact of multipath when using smartphones in forested areas has not yet been thoroughly examined, and that it is essential to investigate the horizontal position accuracy of smartphones in environments where they may often be used [7].

The first objective of this study was to evaluate the feasibility of three GNSS receivers (a smartphone, a Garmin receiver, and a Trimble receiver) as reliable GNSS receivers in a forested area, by comparing their positional accuracy. This study focused on the practical common use of GNSS receivers in forestry by limiting the observation time (less than a minute) during the field data collection effort. The performance of the GNSS receivers was evaluated based on the static horizontal positional accuracy that practitioners regularly encounter, employing neither post-process algorithms nor external GNSS antennas. The Trimble receiver was evaluated in this study as the control, the type of receiver that professional foresters commonly use for navigation and field data collection activities. The second objective of this study was to investigate the impact of nearby forest conditions on the positional accuracy of these GNSS receivers. A suite of forest variables that were based on the location and size of nearby trees was developed. Some of these forest variables were guided by Tobler’s First Law that states “near things are more related than distant things” [26]. We hypothesized that forest vegetation, as described by the size and location of nearby trees, is correlated with the observed horizontal position error one may experience when determining positions with a GNSS receiver. We, therefore, investigated whether the distance or direction of trees to a sample point, along with the size of the trees, had an influence on the horizontal accuracy of positions determined by GNSS receivers.

This article is structured as follows: the Materials and Methods section describes the GPS test site and how the reference locations were established. This section also explains how the forest variables were measured and developed to examine potential correlations with positional accuracy. Moreover, the characteristics of GNSS receivers that were used to locate the positions and their measurements are described. The statistical methods to evaluate positional accuracy and to examine correlation are also explained in this section. In the Results section, the observed positional accuracy of GNSS receivers and the correlations between forest variables and positional accuracy are presented. Lastly, in the Discussion section, the limitations of this study and a suggestion for future research are proposed.

## Materials and methods

The control points for this study were established based on the surveyed control points at the Whitehall Forest GPS Test Site (gps-test-site.uga.edu) in Athens, Georgia (USA). As this site was established by University of Georgia for research purposes, no permit was required for this study. The GPS Test Site contains three Online Positioning User Service (OPUS) monuments and 37 permanent control points which were established in 2004 by professional surveyors. The positions of the three monuments were determined using positions (epochs, position fixes) collected for 4 hours with an Ashtech Locus survey-grade GNSS receiver. As reported by the surveyors, the *X*, *Y*, and *Z* accuracies were (0.009 m, 0.007 m, 0.004 m), (0.079 m, 0.053 m, 0.063 m), and (0.038 m, 0.030 m, 0.034 m), respectively. The final adjustment and horizontal positional precision of these three OPUS monuments was noted by the surveyors to be less than 2 cm, and they have been accepted into the National Spatial Reference System (NSRS). These monuments are marked with aluminum pins about 9 cm in diameter and 1 m long. They are embedded in the ground with the top of the pin flush with the ground surface. The horizontal positions of the 37 permanent control points throughout the GPS Test Site were determined using a total station (Topcon GTS-211D) instrument to develop a closed traverse survey based on the three OPUS monuments. The horizontal positions of these 37 permanent control points are considered to be nearly as accurate as those of the three OPUS monuments.

Two of the 37 permanent control points were selected and used as centers around which we established several other temporary control points. These two permanent control points were located within an older, deciduous, uneven-aged forest consisting of *Quercus* spp., *Carya* spp., *Ostrya virginiana*, and other tree species, of which the density was 126 trees ha^-1^, the basal area was 23.0 m^2^ ha^-1^, and the dominant trees were 70 to 80 years old. We have noted in prior research [27, 28] that the type of forest can be influential on the positional accuracy observed; thus, this research concerns positional accuracy in an eastern U.S. deciduous forest.

**Fig 1:**
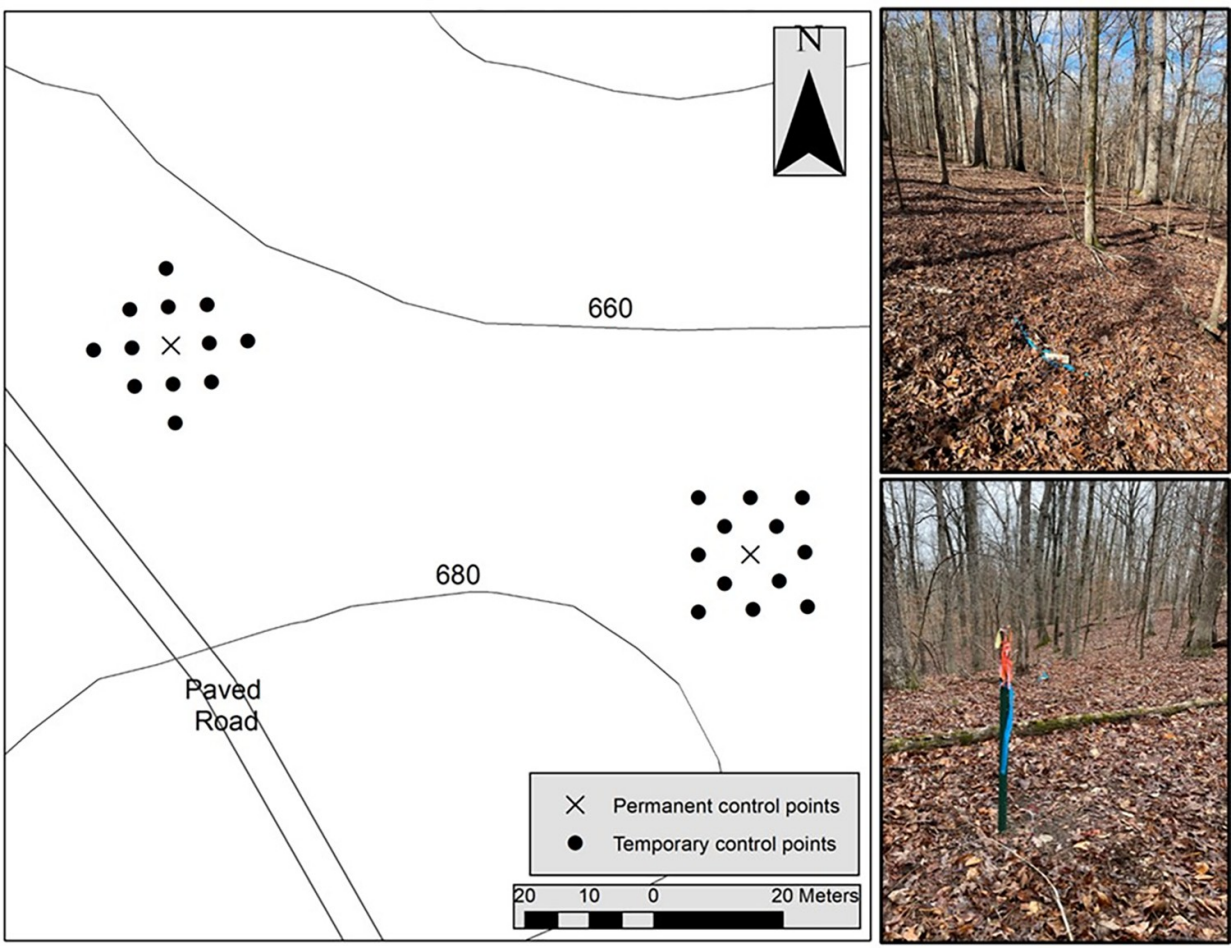
The study location of a portion of the GPS Test Site in Athens, Georgia (USA). The temporary control points were placed on a 6 m grid around a permanent control point.

The environmental conditions around two permanent control points were similar, each situated within a mixed hardwood forest without understory vegetation. These control points were located near the top of a local ridge, with a northerly aspect where the ground slope was less than 7%. As the control points were located in close proximity, the sky view was relatively the same for each, although the positions of nearby trees varied. Around the two permanent control points, 24 temporary control points were established, providing a total of 26 control points upon which this study is based (Fig 1). Each temporary control point was located on a 6 m grid around one of the two permanent control points, up to a radius of 12 m (Fig 1). To establish the position of the temporary control points, straight lines between the positions of other permanent control points and the two which was selected for this research were carefully installed using string, and perpendicular distances from these lines to establish the temporary control points were measured. To augment the establishment of these temporary control point positions, a fixed distance from 45° angles to nearby permanent and temporary control points was also measured. Therefore, based on the positional information of surveyed points, the location of temporary control points using triangulation was calculated.

Three different GNSS receivers were used in this research study to evaluate horizontal position accuracy and tree interactions. The Trimble Juno T41/5 receiver (Trimble Inc., Sunnyvale, CA, USA) was equipped with an L1 GPS antenna that was manufactured by Inpaq technology Co. Ltd (Chunan, Miaoli, Taiwan) (70×43.18×9 mm). Trimble receivers have been shown to provide very good horizontal position accuracy (1 to 5 m) under forest canopies [29–31]. With this GNSS receiver, positional data were collected using SOLO Forest software [31] which allows users to change settings such as the maximum PDOP (positional dilution of precision), minimum SNR (signal to noise ratio), and whether the Wide Area Augmentation System (WAAS) is used. For this study, the maximum PDOP was assumed to be 8, the minimum SNR was assumed to be 4, and WAAS augmentation was enabled. Signals from both the US NAVSTAR GPS program and the Russian GLONASS program were used to determine horizontal positions, although we were unable to know how many of each were used at any one specific point in time, as this information was not reported by the receiver. Unlike other GNSS receivers we tested, the Trimble GNSS receiver collected points (position fixes) and averaged those to determine a single horizontal position and allowed the user to monitor the standard deviation of the location of the determined positions and the number of position fixes that were recorded. The final, averaged determined position was accepted as a data observation only when the standard deviation was less than 1.5 meters, and the number of position fixes was greater than 25 (about 30 seconds). The data was recorded using the WGS84 coordinate system and exported in a shapefile format for further processing.

An additional GNSS receiver, the Garmin Oregon 700 GNSS receiver (Garmin, Olathe, KS, USA), and a versatile smart device, an iPhone 12 Pro smartphone (Apple Inc., Cupertino, CA, USA), were also used in this study. Compared to a Trimble receiver, these GNSS receivers did not allow specific PDOP and SNR settings. Further, these receivers determined positions using a single point (position fix) rather than the average of multiple points. Therefore, each location was determined by averaging ten points (fixes) with 3-second intervals between measurements. The Garmin receiver is a traditional handheld GNSS receiver with a GPS antenna made by Cirocomm Technology Corp. (Taoyuan City, Taiwan) (size: 15×15×4 mm). It allows users to choose which satellite constellation(s) (NAVSTAR GPS and GLONASS) to use and to enable WAAS augmentation. Therefore, we used NAVSTAR GPS and GLONASS in this study. The Garmin receiver had the ability to determine positions through the averaging of multiple position fixes, but this function and WAAS augmentation were not enabled in this study as they could not be monitored. Data was exported from the Garmin receiver to an Excel file format using Basecamp software (Garmin, Olathe, KS, USA). Regarding the iPhone 12 Pro, multiple antennas (12) are equipped in the unit, and each has a specific purpose, such as capturing ultra-wide band (UWB) and ultra-high bend (UHB) signals from 5G networks, along with Wi-Fi, cellular, GNSS, or Bluetooth signals. The iPhone 12 Pro can utilize dual frequency GNSS signals, including L1 and L5 for acquiring positional information, using two different antennas equipped at the top and bottom of the unit. The multiple antennas are part of one system, so it was difficult to measure specific size of antennas for capturing L1 and L5 signals precisely. The antennas used to collect L1 and L5 signals are very small (around 10×10 mm). According to the manufacturer’s manual, the iPhone can utilize satellite signals from a wide collection of satellite systems, including NAVSTAR GPS, GLONASS, Galileo, QZSS, and BeiDou. However, the iPhone 12 Pro does not offer access to the raw GNSS measurements; thus, information such as carrier frequency and noise level were not available. The data was collected using the Avenza Maps application (Avenza System Inc., Toronto, Canada). This application allows users to collect waypoints by tapping the screen of the smartphone. Data is exported to a Keyhole Markup Language (KML) file. Both the Garmin receiver and smartphone recorded data using the WGS84 coordinate system. After data collection was complete, data collected by each GNSS receiver was projected using NAD 83/UTM 17N for further processing.

Each of the 26 control points was visited 20 times during the leaf-off season (February and March 2021). The order of visit was randomized each time. At a visit to a control point, the order of the data collection effort was also randomized for the three GNSS receivers. A leveling monopod was used to place GNSS receivers on top of each control point and to maintain a constant position during the data collection effort. Data were collected during similar time ranges each day to have similar satellites distributed around sky, and the researcher always stood on the north side of monopod when data was being collected. An attempt to collect data at exact sidereal time was not employed as other work [32] suggests this effort may be moot in forests when there is no control over the use of satellite signals by GNSS receivers. Even though we made efforts to reduce any potential effects induced by accepting signals from different satellites or constellations for every visit, the effects could not be removed. The GNSS receivers utilized in this study could not be modified to receive signals from specific satellites. Further, collecting spatial data at the same location simultaneously using three GNSS receivers was not possible when the data collection processes were randomized. So, we changed GNSS receivers promptly to reduce related time delay.

The horizontal accuracy of GNSS receivers was evaluated using the root mean square error (RMSE) of the determined positions, as compared to the true positions of the control points, an assessment commonly employed in this type of study (i.e., [27, 33, 34]).


$$RMSE=\sqrt{\frac{\sum_{i}^{n}\left({\left(x_{i}-x\right)}^{2}+{\left(y_{i}-y\right)}^{2}\right)}{n}}$$

Here, *n* is the total number of observations in a visit; *i* is the *i*th observation of the visit; *x*_*i*_ and *y*_*i*_ are the easting and northing, respectively, of the *i*th observations; and *x* and *y* are the assumed true easting and northing of the associated control point.

**Fig 2:**
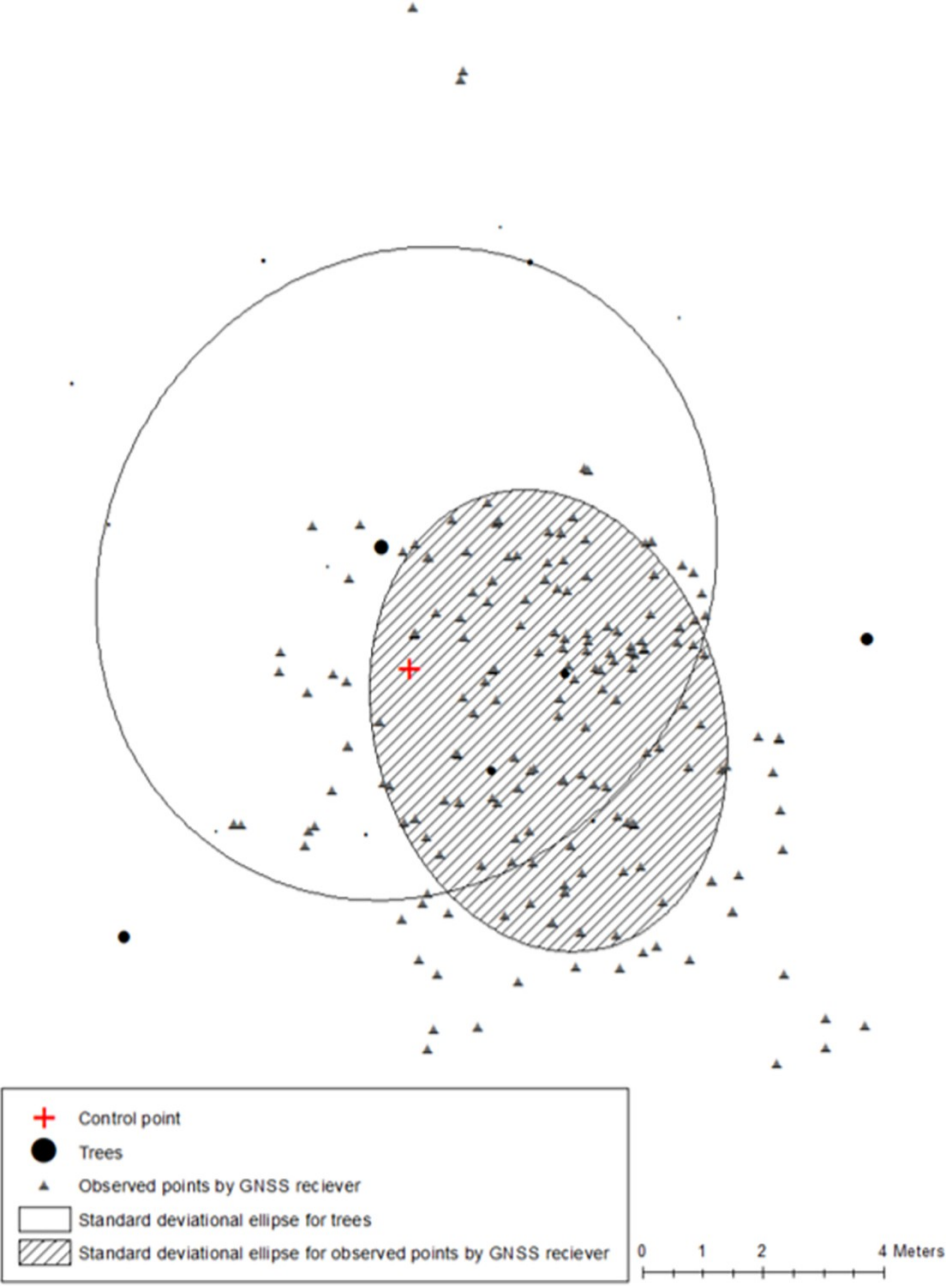
An example of standard deviational ellipses. Each standard deviational ellipse was determined based on the location of nearby trees and the observed points by GNSS receiver.

Standard deviational ellipses were applied to quantify the directional distribution of determined positions in regard to a related control point (Fig 2). The results of this analysis provide the angle (directional distribution), area (distribution), and the mean center (centroid of ellipse representing center tendency) which is equivalent to the root squared error of the mean (RSEM) [33].

To obtain the forest variables, every tree greater than 1.37 m tall, which is equal to the height of a monopod, either dead or alive, was measured if it was located within 20 m from one of the two permanent control points. The location of each tree’s stem was determined using a closed traverse survey by measuring distances and azimuth between trees and temporary and permanent control points. Specifically, the azimuth was measured from a control point to the center of stem using a laser rangefinder (TruPulse 360R, Laser Technology Inc., Centennial, CO, USA). Further, half of the diameter at breast height (DBH) was added to the measured distance to estimate the location of stem center. Each control point (permanent and temporary) was assumed as a center of a plot having a radius of 8 m, which is equivalent a 0.02 ha (0.05 acre) plot, resulting in 26 plots with different groups of nearby trees. Forest condition variables were summarized only for those trees within 8 m from each control point. Thirty-five forest condition variables were developed for assessing the correlation between condition and the observed horizontal position error of the GNSS receivers (Table 1). Since the data was normally distributed, multivariate regression analysis using R (version 3.6.1, RStudio, Inc., Boston, MA, USA) was conducted to evaluate the association of forest condition variables and horizontal position error observed by the GNSS receivers.

**Table 1:** A summary of forest variables used in the correlation analysis.

Forest variables	Description
1. Tree count	The total number of trees (living and dead) within 8 m or less from a control point
2. Average distance to trees	The average distance (m) between a control point and the center of trees 8 m or less from the control point
[Ave. Dist.]	
3. Average DBH	The average DBH (cm) of trees 8 m or less from a control point
[Ave. DBH]	
4. Standard deviation of DBH	The standard deviation of the DBH of trees 8 m or less from a control point
[SD. DBH]	
5. Coefficient of variation of DBH	The coefficient of variation of the DBH of trees 8 m or less from a control point
[CV. DBH]	
6. Average basal area of trees	The average basal area (m^2^) of trees 8 m or less from a control point
[Ave. BA]	
7. Standard deviation of basal area	The standard deviation of the basal area of trees 8 m or less from a control point
[SD. BA]	
8. Coefficient of variation of basal area	The coefficient of variation of the basal area of trees 8 m or less from a control point
[CV. BA]	
9. Distance to the mean center of an ellipse formed by nearby trees	The distance (m) from a control point to the mean center of the ellipse formed by trees 8 m or less from the control point
[Dist. MC]	
10. Latitude (north-south) distance to the mean center of an ellipse formed by nearby trees	The difference (m) in the *Y* coordinate of the control point and the *Y* coordinate of the mean center of the ellipse formed by trees 8 m or less from the control point
[Lat. MC]	
11. Departure distance to the mean center of an ellipse formed by nearby trees	The difference (m) in the *X* coordinate of the control point and the *X* coordinate of the mean center of the ellipse formed by trees 8 m or less from the control point
[Dep. MC]	
12. Distance to the mean center of an ellipse formed by nearby trees, weighted by DBH	The distance (m) from a control point to the mean center of the ellipse formed by trees 8 m or less from the control point, weighted by the DBH of those trees
[Dist. MC_DBH_]	
13. Latitude (north-south) distance to the mean center of an ellipse formed by nearby trees, weighted by DBH	The difference (m) in the *Y* coordinate of the control point and the *Y* coordinate of the mean center of the ellipse formed by trees 8 m or less from the control point, weighted by the DBH of those trees
[Lat. MC _DBH_]	
14. Departure (east-west) distance to the mean center of an ellipse formed by nearby trees, weighted by DBH	The difference (m) in the *X* coordinate of the control point and the *X* coordinate of the mean center of the ellipse formed by trees 8 m or less from the control point, weighted by the DBH of those trees
[Dep. MC _DBH_]	
15. Distance to the mean center of an ellipse formed by nearby trees, weighted by basal area	The distance (m) from a control point to the mean center of the ellipse formed by trees 8 m or less from the control point, weighted by the basal area of those trees
[Dist. MC_BA_]	
16. Latitude (north-south) distance to the mean center of an ellipse formed by nearby trees, weighted by basal area	The difference (m) in the *Y* coordinate of the control point and the *Y* coordinate of the mean center of the ellipse formed by trees 8 m or less from the control point, weighted by the basal area of those trees
[Lat. MC _BA_]	
17. Departure (east-west) distance to the mean center of an ellipse formed by nearby trees, weighted by basal area	The difference (m) in the *X* coordinate of the control point and the *X* coordinate of the mean center of the ellipse formed by trees 8 m or less from the control point, weighted by the basal area of those trees
[Dep. MC _BA_]	
18. Latitude (north-south) distance to the largest tree	The difference (m) in latitude between the control point and the largest tree within 8 m of the control point
[Lat. Tree_1_]	
19. Departure (east-west) distance to the largest tree	The difference (m) in departure between the control point and the largest tree within 8 m of the control point
[Dep. Tree_1_]	
20. Largest tree basal area / distance to the largest tree	The basal area (m^2^) of the largest tree within 8 m of a control point divided by the distance (m) to this largest tree from the control point
[BA/Dist. Tree_1_]	
21. Largest tree basal area / latitude (north-south) distance to the largest tree	The basal area (m^2^) of the largest tree within 8 m of a control point divided by the difference (m) in latitude between the control point and the largest tree
[BA/Lat. Tree_1_]	
22. Largest tree basal area / departure (east-west) distance to the largest tree	The basal area (m^2^) of the largest tree within 8 m of a control point divided by the difference (m) in departure between the control point and the largest tree
[BA/Dep. Tree_1_]	
23. Average of two largest tree basal areas / average distance to the two largest trees	The average basal area (m^2^) of the 2 largest trees within 8 m of a control point divided by the average distance (m) to the 2 largest trees from the control point
[BA/Dist. Tree_2_]	
24. Average of two largest tree basal areas / average latitude (north-south) distance to the two largest trees	The average basal area (m^2^) of the 2 largest trees within 8 m of a control point divided by the average latitude distance (m) to the 2 largest trees from the control point
[BA/Lat. Tree_2_]	
25. Average of two largest tree basal areas / average departure (east-west) distance to the two largest trees	The average basal area (m^2^) of the 2 largest trees within 8 m of a control point divided by the average departure distance (m) to the 2 largest trees from the control point
[BA/Dep. Tree_2_]	
26. Average of three largest tree basal areas / average distance to the three largest trees	The average basal area (m^2^) of the 3 largest trees within 8 m of a control point divided by the average distance (m) to the 3 largest trees from the control point
[BA/Dist. Tree_3_]	
27. Average of three largest tree basal areas / average latitude (north-south) distance to the three largest trees	The average basal area (m^2^) of the 3 largest trees within 8 m of a control point divided by the average latitude distance (m) to the 3 largest trees from the control point
[BA/Lat. Tree_3_]	
28. Average of three largest tree basal areas / average departure (east-west) distance to the three largest trees	The average basal area (m^2^) of the 3 largest trees within 8 m of a control point divided by the average departure distance (m) to the 3 largest trees from the control point
[BA/Dep. Tree_3_]	
29. Average (basal area)^2^	The average basal area (m^2^) of trees 8 m or less from a control point, squared
[Ave. BA^2^]	
30. Average (latitude × (basal area)^2^)	The average of the latitude (north-south) distance (m) from each tree within 8 m of a control point to the control point, times the basal area (m^2^) squared of the tree
[Ave. Lat. BA^2^]	
31. Average (departure × (basal area)^2^)	The average of the departure (east-west) distance (m) from each tree within 8 m of a control point to the control point, times the basal area (m^2^) squared of the tree
[Ave. Dep. BA^2^]	
32. Ellipse area	The average area (m^2^) of the standard deviational ellipse formed by trees within 8 m of a control point
[Area _tree_]	
33. Overlapping ellipse area	The area of overlap (m^2^) between the ellipse formed by trees within 8 m of a control point, and the ellipse formed by the observed positions from a GNSS receiver at that same control point
[Overlap Area]	
34. Overlapping ellipse percent from the perspective of the observed GNSS positions	The area of overlap (m^2^) between the ellipse formed by trees within 8 m of a control point, and the ellipse formed by the observed positions from a GNSS receiver at that same control point, divided by the size of the ellipse formed by the observed positions from a GNSS receiver at that same control point
[Overlapped Area _GNSS receiver_]	
35. Overlapping ellipse percent from the perspective of the trees within 8 m of a control point	The area of overlap (m^2^) between the ellipse formed by trees within 8 m of a control point, and the ellipse formed by the observed positions from a GNSS receiver at that same control point, divided by the size of the ellipse formed by the trees within 8 m of that same control point
[Overlapped Area _tree_]	

## Results

**Fig 3:**
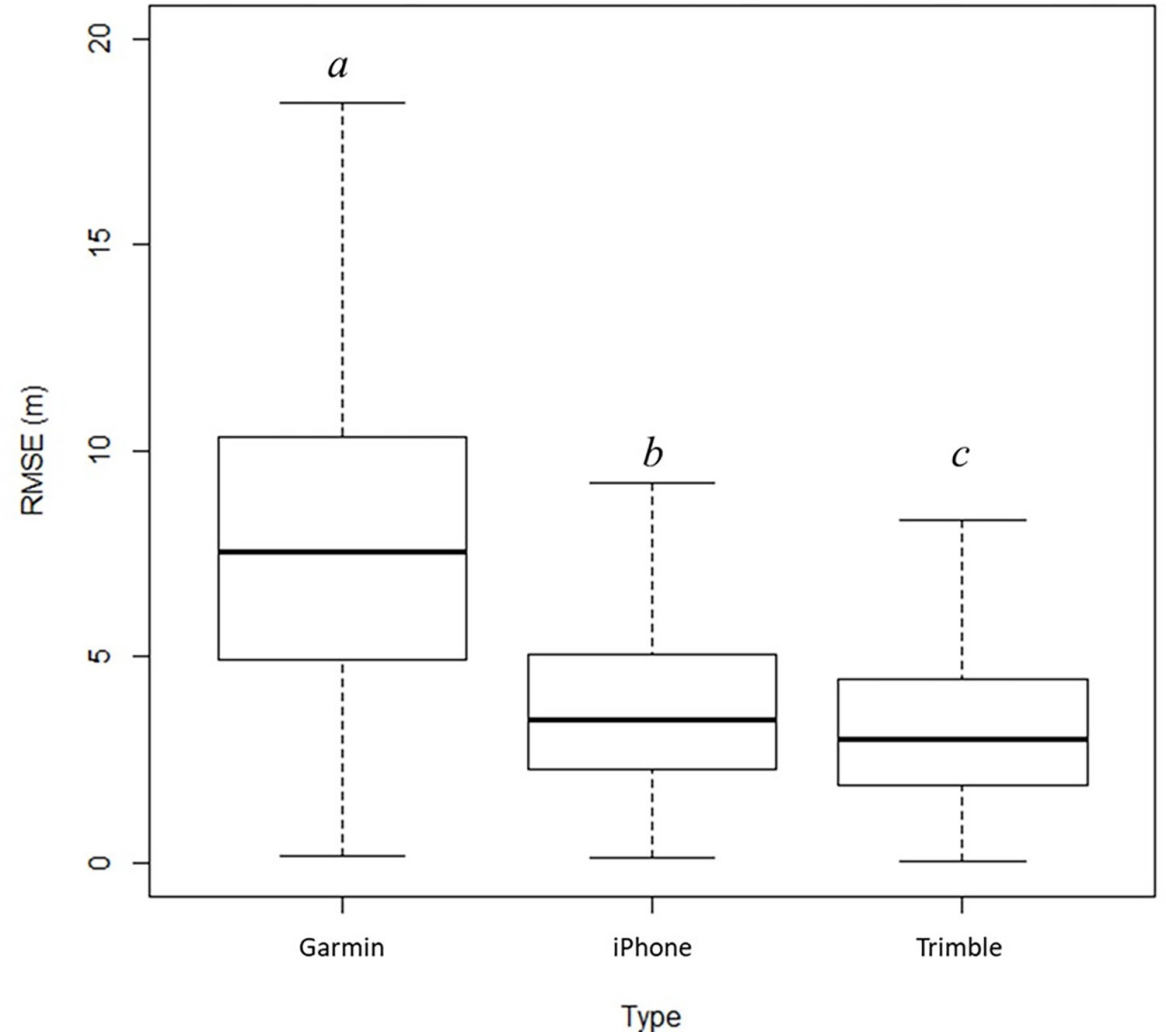
Boxplot for horizontal positional accuracy (RMSE, root mean square error) by GNSS receiver types. A *different letter* indicates statistically significant differences (*p*-value < 0.001).

Within 8 m of each control point, we found an average number of 14.5 trees and an average DBH of 17.0 cm (Table 2). Within these plots, the average distance of trees from each control point ranged from 4.60 to 6.03 m, with an overall average of 5.31 m. Although somewhat variable, more trees seemed to be located slightly to the West and South directions from each control point, as evidenced by the negative average departure (east-west difference) and negative average latitude (north-south difference) in Table 2. The average horizontal positional error (RMSE) observed by the GNSS receivers, for each control point, ranged from 2.28 to 9.77 m (Table 3). The minimum and maximum average RMSE values for the Garmin receiver were 5.75 m and 9.77 m. When the smartphone was used, the minimum and maximum average RMSE were 3.04 m and 5.64 m. With the Trimble receiver, the minimum and maximum average RMSE were 2.28 m and 6.19 m. In general, the smallest average positional error was obtained using the Trimble receiver; however, the performance of the smartphone was very similar in this respect. The largest average positional error was obtained using the Garmin receiver. The average RMSE from every control point determined by the Garmin receiver, smartphone, and Trimble GNSS receiver was 8.11 m (± 4.61 m), 3.91 m (± 2.36 m), and 3.45 m (± 2.18 m), respectively (Fig 3). From this information, we observed a significant difference in the average RMSE by GNSS receiver type.

**Table 2:** A summary of tree characteristics within 8 m of each control point.

Control point	Number of trees	Average of distance (m)	Average DBH (cm)	Average of departure (m)	Average of latitude (m)
Every control point	14.5	5.31	17.0	-0.05	-0.10
1	15	5.17	18.3	-0.26	-0.43
2	10	5.23	19.1	0.50	1.41
3	14	6.03	18.2	-1.05	-0.21
4	11	4.73	11.4	0.34	1.41
5	11	5.90	19.3	-0.20	-1.37
6	11	4.75	20.2	0.94	-0.10
7	10	5.23	18.8	1.02	-0.51
8	12	5.32	16.3	0.01	1.10
9	19	5.56	15.3	0.03	2.06
10	14	5.43	19.3	0.09	-0.09
11	12	5.63	19.0	-0.10	0.23
12	15	5.05	16.0	0.64	0.25
13	15	5.71	20.1	0.32	-0.00
14	14	4.84	17.0	0.00	-0.42
15	17	4.82	12.4	0.42	-0.99
16	18	5.98	18.5	0.53	0.13
17	18	5.10	15.0	-1.02	0.98
18	16	5.31	12.3	-0.63	0.99
19	17	4.99	14.3	0.33	-0.09
20	17	5.79	20.0	0.55	-0.37
21	18	5.37	20.0	-0.63	0.16
22	18	5.39	13.7	-1.11	-1.88
23	15	5.06	13.9	0.06	-1.58
24	17	5.65	19.5	-0.31	-0.60
25	12	4.60	19.4	-1.78	-0.96
26	11	5.39	15.7	-0.08	-1.83

**Table 3:** The positional error (root mean square error, RMSE) and the area of standard deviational ellipses observed by different GNSS receivers at each control point.

Control point	RMSE (m)			Area of standard deviational ellipses (m^2^)			
	*Garmin Oregon 700*	*iPhone 12 Pro*	*Trimble Juno T41*		*Garmin Oregon 700*	*iPhone 12 Pro*	*Trimble Juno T41*	
1	7.99	3.56	3.20		173.75	34.44	27.77	
2	7.55	3.52	3.47		113.17	30.58	33.00	
3	8.40	4.32	2.55		158.84	49.23	23.30	
4	8.93	3.79	4.08		164.55	48.46	43.62	
5	8.76	3.21	4.22		159.56	23.83	54.06	
6	8.57	3.82	3.04		148.69	47.90	27.15	
7	9.17	4.41	3.65		134.44	34.96	31.69	
8	5.91	3.42	3.75		132.12	30.66	44.66	
9	5.75	3.04	3.17		107.74	32.75	36.44	
10	9.21	4.14	4.46		107.31	49.19	46.20	
11	6.89	3.74	3.60		107.78	33.09	42.34	
12	9.23	4.24	3.32		190.69	53.32	39.46	
13	7.53	3.20	3.32		131.01	22.57	34.68	
14	7.68	3.52	6.19		257.47	42.20	50.17	
15	7.29	3.26	3.23		169.93	36.35	33.59	
16	7.72	3.95	2.79		125.35	41.56	26.32	
17	7.86	4.17	3.97		192.17	33.52	64.14	
18	8.76	3.46	2.98		189.15	36.54	37.55	
19	8.70	4.32	3.00		174.13	49.93	34.58	
20	8.54	4.03	2.96		142.38	51.55	28.66	
21	6.95	3.56	2.72		140.27	32.51	27.45	
22	8.67	4.86	3.21		226.93	58.52	42.30	
23	8.37	3.78	3.34		171.95	35.35	48.04	
24	8.33	4.08	3.23		158.38	42.78	47.04	
25	9.77	4.76	2.28		221.55	52.88	19.85	
26	8.27	5.64	4.01		421.08	63.29	45.77	

**Fig 4:**
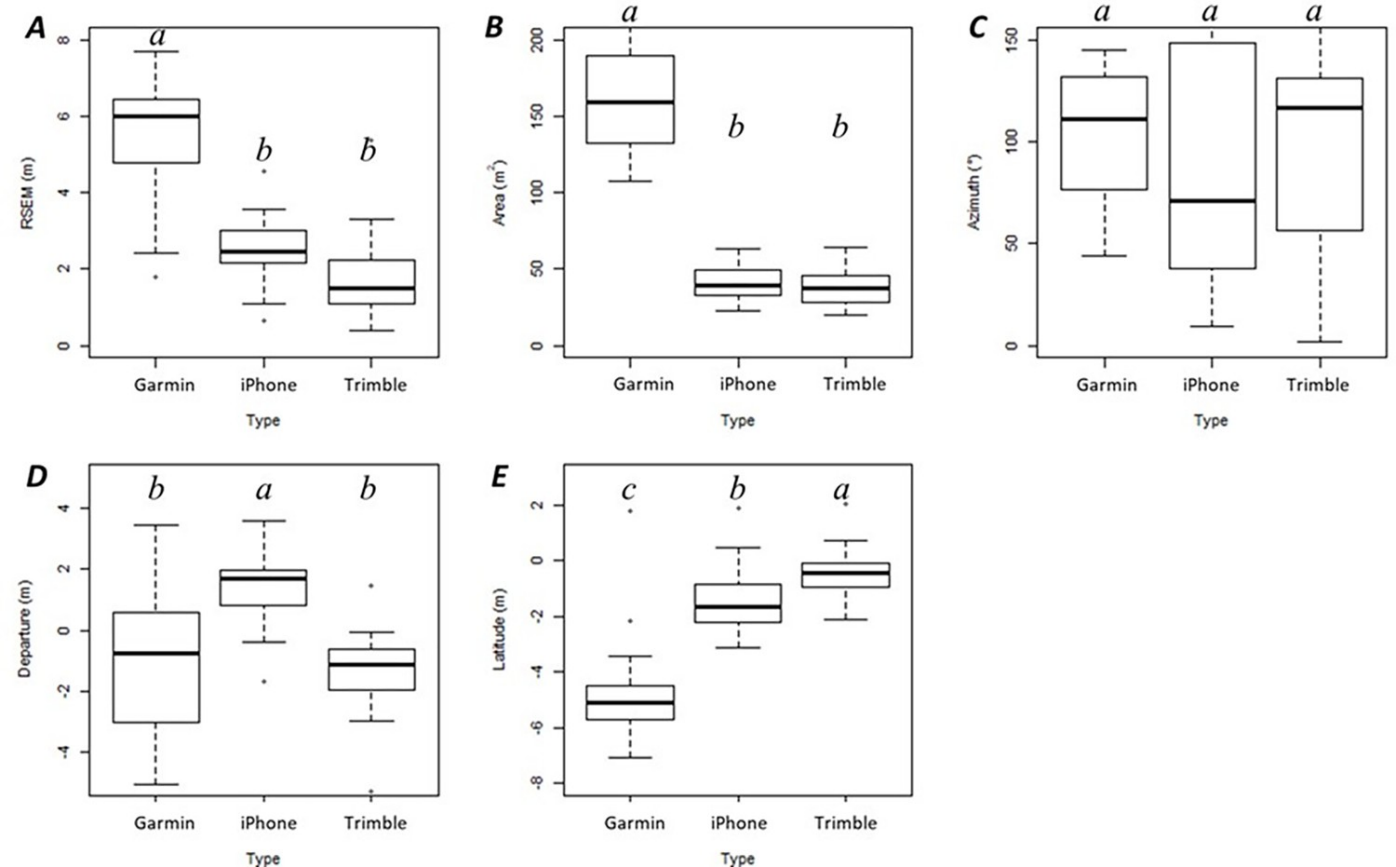
Boxplot for directional distribution observed by GNSS receivers (*A*: RSEM (root squared error of the mean, calculated based on the mean center); *B*: Area of ellipse (m^2^); *C*: Rotation (°); *D*: Departure (m); *E*: Latitude (m)). A *different letter* indicates significant differences (*p* < 0.05).

Parameters related to the distribution of observed points also indicated some significant differences between GNSS receivers (Fig 4). For example, the RSEM indicating the distance between mean center of observed GNSS-determined positions and related control points and the area of standard deviational ellipse for GNSS-determined positions suggested no significant differences were observed between the smartphone and the Trimble receiver. However, the RSEM and standard deviational ellipse area for observed GNSS-determined positions using the Garmin receiver were significantly different from both of the other devices. The larger area of standard deviational ellipses from the observed GNSS-determined positions of the Garmin receiver suggested that the observed points were more likely distributed wider around the control point. This also suggested that the Garmin receiver had a larger variance in positional error.

We observed no statistically significant difference in the rotation representing the directional distribution of the observed GNSS positions, regardless of the GNSS receiver types due to its wide variance (Table 4, Fig 4). This meant that the directional distribution might be the most susceptible parameter that could be influenced by environmental factors regardless of GNSS receivers. The average departure was negative for observed GNSS positions collected by the Garmin and Trimble receivers (Table 4) indicating observed positions were often slightly (about 1 m) to the West of the control points. When the smartphone was used, the average departure was positive meaning that the mean center for the distribution of observed GNSS positions was located East (about 1.5 m) of each control point. Otherwise, the average latitude was negative regardless of GNSS receiver, indicating that the mean center for the distribution of observed GNSS positions was South of each control point, nearly 5 m South on average in the case of the Garmin receiver.

**Table 4:** The summary of elliptical metrics from measurements obtained by different GNSS receivers.

Metric		*Garmin Oregon 700*	*iPhone 12 Pro*	*Trimble Juno T41*
*RSEM* (m)				
	Average	5.51	2.52	1.75
	St. deviation	1.42	0.79	1.06
	Minimum	1.78	0.64	0.40
	Maximum	7.70	4.55	5.35
*Area* (m^2^)				
	Average	170.01	41.08	38.07
	St. deviation	63.73	10.57	10.44
	Minimum	107.31	22.57	19.85
	Maximum	421.08	63.29	64.14
*Rotation* (°)				
	Average	105.59	87.99	97.54
	St. deviation	29.08	50.97	57.93
	Minimum	44.10	9.23	2.10
	Maximum	144.79	170.67	167.23
*Departure* (m)				
	Average	-0.86	1.49	-1.29
	St. deviation	2.36	1.15	1.28
	Minimum	-5.05	-1.69	-5.27
	Maximum	3.42	3.58	1.46
*Latitude* (m)				
	Average	-4.84	-1.47	-0.44
	St. deviation	1.68	1.16	0.86
	Minimum	-7.06	-3.15	-2.10
	Maximum	1.77	1.90	2.05

RSEM—root squared error of the mean; Area—the area of standard deviational ellipse; Rotation—the directional distribution of observed points; Departure—the difference between observed X coordinates and control X coordinate; Latitude—the difference between observed Y coordinates and control Y coordinate.

Standard deviational ellipses were modeled for observed GNSS-determined positions at each control point, and even though the average size (area) of these was not statistically significant between the smartphone and the Trimble receiver, statistically significant differences in the size of these ellipses were observed between the Garmin receiver and the other two receivers. Not surprisingly, the maximum area of an ellipse at any one control point (about 421 m^2^) was observed using data collected by the Garmin receiver (Table 3). The minimum ellipse area (about 20 m^2^), indicating high precision amongst observed GNSS-determined positions, was observed by the Trimble receiver.

The correlations between positional error (RMSE) and forest variables indicated that in general there was a weak to moderate correlation between these regardless of GNSS receiver employed (Table 5). However, two forest variables indicated a significant correlation with positional error with all three GNSS receivers. First, the difference between the *X* coordinate of a control point and the *X* coordinate of the mean center of the ellipse formed by trees 8 m or less from the control point, weighted by the DBH of those trees, was statistically significant and positively correlated with error in positions determined by the smartphone and the Garmin receiver, and statistically significant and negatively correlated with error in positions determined by the Trimble receiver. Second, the area of overlap between the ellipse formed by trees within 8 m of a control point, and the ellipse formed by the observed positions from a GNSS receiver at that same control point, divided by the size of the ellipse formed by the observed positions from a GNSS receiver at that same control point, was statistically significant and negatively correlated with error in positions determined by all three devices. In this latter case, as the overlapping area of the ellipse formed by trees within 8 m of a control point and the ellipse formed by GNSS-determined positions increased, positional error decreased. The location of nearby trees with respect to positions being determined, either shifted away (low overlap of ellipses) or directly in the vicinity (high overlap in ellipses), does seem to affect positional error. Further, in this latter case, the strongest correlation was observed amongst the positions determined by the three devices.

**Table 5:** A summary of Pearson correlation coefficients for RMSE and forest variables (†*p*-value < 0.05; ‡ *p*-value < 0.01; ⁂*p*-value <0.001).

Forest variable	*Garmin Oregon 700*	*iPhone 12 Pro*	*Trimble Juno T41*
Tree count	-0.26	-0.13	-0.28
Ave. Dist.	-0.40	-0.08	-0.17
Ave. DBH	0.00	-0.04	-0.11
SD. DBH	-0.10	0.11	-0.12
CV. DBH	-0.13	0.13	-0.02
Ave. BA	-0.13	-0.14	-0.22
SD. BA	-0.01	0.07	-0.18
CV. BA	0.20	0.47 †	0.05
Dist. MC	0.05	0.27	-0.17
Lat. MC	0.45 †	0.50 ‡	0.01
Dep. MC	0.08	0.26	-0.20
Dist. MC_DBH_	-0.24	0.17	0.16
Lat. MC _DBH_	0.41 †	0.27	0.09
Dep. MC _DBH_	0.58 ‡	0.45 †	-0.46 †
Dist. MC_BA_	-0.14	-0.06	0.15
Lat. MC _BA_	0.39 †	0.14	0.07
Dep. MC _BA_	0.61 ⁂	0.34	-0.37
Lat. Tree_1_	-0.42 †	-0.21	-0.03
Dep. Tree_1_	-0.24	-0.13	0.05
BA/Dist. Tree_1_	-0.12	-0.28	-0.10
BA/Lat. Tree_1_	-0.04	0.56	0.03
BA/Dep. Tree_1_	-0.14	0.07	0.58 ‡
BA/Dist. Tree_2_	-0.23	0.03	-0.14
BA/Lat. Tree_2_	0.21	0.13	0.24
BA/Dep. Tree_2_	-0.17	-0.29	0.22
BA/Dist. Tree_3_	-0.28	-0.10	0.18
BA/Lat. Tree_3_	0.06	-0.18	0.20
BA/Dep. Tree_3_	0.01	0.04	0.46 †
Ave. BA^2^	0.01	0.03	-0.21
Ave. Lat. BA^2^	-0.22	-0.18	-0.09
Ave. Dep. BA^2^	-0.38	-0.19	0.16
Area _tree_	-0.13	0.04	-0.03
Overlapped Area	-0.70 ⁂	0.20	0.10
Overlapped Area _GNSS receiver_	-0.88 ⁂	-0.68 ⁂	-0.85 ⁂
Overlapped Area _tree_	-0.59 ‡	0.12	0.07

BA—Basal area

DBH—Diameter at breast height

Dep—Departure, or east-west difference from true control point position

Dist—Distance from true control point position

MC—The mean center of objects

Lat—Latitude, or north-south difference from true control point position

However, different trends in statistically significant correlations were observed between the three devices. For instance, nine of the forest variables were found to be significantly correlated with the positional error inherent in the data collected by the Garmin receiver, and four forest variables were found to be significantly correlated with the positional error inherent in the data collected by the smartphone and the Trimble receiver. These results indicated that the horizontal position error of the Garmin receiver was more likely affected by the forest variables we measured than the smartphone and the Trimble receiver.

## Discussion

Based on the results of our study, the positional data collected by the smartphone had a similar level of horizontal positional accuracy and variance in forested conditions as compared to a Trimble receiver, even though it technically is not a traditional type of GNSS receiver. The horizontal positional accuracy of the smartphone in this study fell into the range of accuracy (1–12 meters) obtained from previous studies involving smartphones, but again our study indicated that sub-meter positional accuracy was not attainable by any of the devices employed in forested conditions. Zhang et al. [19] pointed out that sub-meter positional accuracy was rarely achievable without the use of post-processing and an external antenna to reduce the effects of multipath. But, according to previous studies, even with the aid of post-processing or external antennas, the positional accuracy of a smartphone may be significantly lower than that obtained by higher grade devices under similar conditions [13, 19, 24, 35]. Yet compared to a dedicated GNSS receiver (Garmin receiver), the smartphone had significantly higher positional accuracy with a lower level of variance. And the horizontal position accuracy of the smartphone was similar to the Trimble receiver we employed, which was relevant to the results of previous studies (2–5 meters).

The duty cycling and limitations of GNSS chipsets equipped within smartphones are considered major reasons why smartphones cannot attain the positional accuracy of higher grade GNSS receivers [2, 7, 17]. Duty cycling, the initiation, use, and termination of work on a particular circuit, was implemented in devices such as smartphones to reduce battery usage, but it hinders the continuity of signal accepting [2]. Gogoi et al. [17] confirmed that duty cycling increased the noise observed in GNSS signals, which deteriorates positional accuracy. Fortunately, duty cycling might be disabled in certain recent Android smartphones, and this advancement is expected to lead to an enhanced positional accuracy of smartphones in the future by allowing better continuity of phase measurements [2].

The GNSS chipsets within smartphones and other devices have been developed to address the general need for greater positional accuracy at an affordable cost to the end-user. According to the manufacturer’s manual, it was confirmed that the smartphone we tested (iPhone 12 Pro) could utilize multiple GNSS constellations and frequencies, as could the Trimble receiver we tested. The ability to obtain signals from multiple GNSS constellations may improve positional accuracy by reducing signal search time [2]. However, determining a position through a multi-constellation system may not always guarantee higher positional accuracy because it could induce noise and signals may be deteriorated by a large number of extreme errors [13, 36]. There are two main manufacturers for smartphone GNSS chipsets, the first dual-frequency chipset was released by Broadcom in 2018, and later that same year Qualcomm also released a dual-frequency chipset [7]. The dual-frequency chipset may enhance positional accuracy by allowing the system to compensate for perceived ionospheric effects [7, 13, 34]. However, specific information about GNSS chipset equipped within the iPhone 12 Pro was not available to the public so we could not confirm whether the iPhone 12 Pro benefitted from these advances. The iPhone 12 Pro was equipped with multiple antennas and two of them were designed for accepting satellite signals. These GNSS chipsets were installed at the top and bottom of the smartphone and designed to detect a specific frequency such as L1 and L5. In the past, the GNSS chipset designed for smartphones may have been considered as a limitation [21], yet two GNSS chipsets designed to detect specific frequencies might be helpful in attaining better positional accuracy.

It has been suggested that the GNSS chipset in smartphones may be susceptible to multipath error [7, 13]; therefore, investigating the correlation between horizontal positional accuracy and forest variables (proxies for the juxtaposition of trees with respect to survey points) was necessary to fill a knowledge gap. According to previous studies, horizontal position accuracy can change in response to the forest type within which the technology is being used [13, 33]. In this study, we observed a few significant correlations between forest variables and horizontal position error regardless of GNSS receiver. However, the assumption that the size of nearby tree stems would influence the horizontal positional accuracy of GNSS receivers was not confirmed. With regard to the departure (east-west position) of the mean center weighted by DBH (Dep. MC _DBH_) which had a significant correlation regardless of GNSS receivers, different trends of correlations were observed depending on the type of GNSS receivers, again rendering the hypothesis that the *size* of nearby trees has a consistent, significant effect of positional accuracy of GNSS determined positions moot. Data from the Garmin receiver indicated a positive moderate correlation, meaning that the farther the center of a tree was from a control point, the higher positional error was observed. Otherwise, the Trimble receiver had a negative moderate correlation, indicating the near vicinity of mean center of tree stems induced the greater positional error. However, regardless of GNSS receiver employed, the percentage of overlap area between the ellipses formed by nearby trees and the ellipses formed by GNSS-determined positions (Overlapped Area _GNSS receiver_) was found to be important, thereby may indicate that there is indeed an effect on the positional accuracy of GNSS-determined positions and the *location* of nearby trees.

Even though we developed a number of forest variables based on tree characteristics and their distribution around each control point, we found it complicated to define or generalize trends in correlations between positional accuracy and forest variables. We confirmed that multipath, likely induced by nearby vegetation, deteriorated the horizontal positional accuracy in the forested study area but how it affected positional accuracy could not be generalized [13, 23]. With this work, we also could confirm that the Garmin receiver was more likely affected by the multipath issue, through correlation analysis of the forest variables we developed, compared to the Trimble receiver and the smartphone, simply based on the number of forest variables having a significant correlation with positional error. Interestingly, the smartphone had the same number of forest variables with significant correlations with positional accuracy as the Trimble receiver. This suggests that smartphone GNSS technology may be approaching the technological sophistication (hence accuracy levels) obtainable with much more expensive field data collection equipment.

This study focused on the effect of the horizontal structure of nearby vegetation. As satellite signals emitted from outer space travel through Earth’s atmosphere, the quality of signals may be more affected by the vertical structure of nearby vegetation (the last 30–60 m of travel before being used) which may be related to the shape of tree crowns and tree architecture. As removing multipath errors may be almost impossible in a real environment, a further study to scrutinize the impact of nearby vegetation seems necessary. Our study suggests that the raw GNSS measurements, such as SNR, should be examined, and that new forest variables should be developed in an attempt to help explain the impact of nearby vegetation on horizontal position accuracy of GNSS devices.

## Conclusions

The feasibility of a smartphone (iPhone 12 Pro) for mapping positions within a forested environment was investigated by comparing the horizontal position error with similar data from traditional handheld types of GNSS receivers (Garmin receiver and a Trimble receiver). The results of this study indicated that the positional accuracy of a smartphone falls in the middle point of two different types of GNSS receivers. In other words, the smartphone illustrated significantly lower horizontal position error than a Garmin receiver, but significantly higher positional error than a Trimble receiver. However, the range of horizontal positional error for the smartphone was closer to the range of positional error observed by a Trimble receiver. For mapping purposes, based on the horizontal accuracy observed, the feasibility of using the Trimble receiver and a smartphone in forested conditions, therefore, seems high. For navigational purposes, the feasibility of using the three GNSS receivers we studied also seems high.

Recent advances in GNSS chipset technology, especially for smartphones, might further enhance their positioning performance, but as with all new technology, these issues need to be constantly assessed. In this study, we also investigated the potential correlation between forest variables that might induce multipath error and the observed horizontal position error. We found significant correlations between forest variables and horizontal position error regardless of the GNSS receiver employed, yet more correlations were observed with respect to the horizontal position error of the Garmin receiver. This suggests that the horizontal position error of the Garmin receiver was more likely affected by forest variables than was the horizontal position error of the smartphone and the Trimble receiver. In addition, it was confirmed that the location, but not the size, of nearby trees may be the primary factor in deteriorating the horizontal positional accuracy. Given these results, generalizing the multipath effect of nearby vegetation on horizontal position accuracy might be more complicated than we assumed.
